# Reproduction and Fertility Beliefs, Perceptions, and Attitudes in People Living with HIV

**DOI:** 10.1155/2018/5349793

**Published:** 2018-04-01

**Authors:** Vaidehi Mujumdar, Doris Berman, Katherine R. Schafer

**Affiliations:** ^1^Wake Forest School of Medicine, 1 Medical Center Blvd, Winston-Salem, NC 27157, USA; ^2^Section on Infectious Diseases, Wake Forest University Health Sciences, Winston-Salem, NC, USA

## Abstract

People living with HIV (PLWH) have distinct needs when it comes to reproductive health, specifically regarding fertility, family planning, and pregnancy, and these needs are often complicated by HIV status. While there is ample research that focuses on reproductive health in PLWH through a quantitative lens, there is a lack of research using qualitative methods, namely, the narrative interview model. We searched PubMed and relevant abstracts to identify 72 articles published from 1997 to 2016 that described a qualitative framework for exploring the behaviors and perceptions regarding family planning, abortion, pregnancy, parenthood, fertility, and forced sterility in PLWH. The inclusion criteria initially showed 147 articles, which were further screened to exclude those that did not address fertility and family planning specifically. Our final sample of articles included articles related to qualitative research on reproductive attitudes, beliefs, and behaviors of PLWH. Several of these articles were mixed-methods analyses, but our focus was on the qualitative portion only. Further qualitative works in this area will not only contribute to gaps quantitative research in the field cannot capture by design, but also inform clinical practice, policy, and interventions through systematic, in-depth evaluation.

## 1. Introduction

Qualitative research on reproductive behaviors and perceptions of people living with HIV (PLWH) is lacking compared to quantitative studies. Often in HIV research, qualitative studies are embedded within larger quantitative studies to further elucidate the data of the larger study [1–6].

Theories of reproductive health in HIV populations increasingly appear in the literature on individual and community health, especially in relation to racial and ethnic minority populations and groups that experience significant health disparities. The vast majority of these studies are conducted using traditional, standard quantitative research methodology such as data coding and content analysis [7, 8].

However, there is a dearth of qualitative studies in the United States that explore the reproductive narratives of PLWH. PLWH encounter many challenges in their interactions with reproductive health services, often impacting their pregnancy decisions [9, 10]. In this review, we summarize the existing qualitative literature on reproductive health perceptions and decision-making for PLWH. This article will contribute knowledge to an area of increasing needs for patient care and best practices because PLWH can now engage in healthy, safe sexual relationships and make informed decisions surrounding safe reproduction with decreased risk of HIV transmission.

## 2. Materials and Methods

We identified articles in the English language literature using PubMed using the following MeSH terms:* HIV-positive*,* HIV seropositive*,* reproductive behavior*,* fertility*,* pregnancy, and qualitative*. We also examined the references of articles identified through the selected articles. Though comprehensive, this process was not a formal systematic review. We chose not to conduct a formal systematic review because of the limited amount of qualitative research on reproductive behavior targeted to fertility, family planning (FP), and pregnancy in the United States. Included studies were primarily qualitative or mixed-methods with a qualitative component. Studies could involve behaviorally infected or perinatally infected participants, of both sexes. We focused on findings presented for females and males, but found that there were not as many studies focused on men. We did not limit our results to a particular geographic location, ethnic group, or country.

Because the purpose of this article and in general of qualitative, semistructured interviews is to explore participants' perspectives and experiences, we did not formally grade the strength or weakness of the evidence of the articles synthesized. Below, we summarize the key themes and findings across the identified articles, each in three primary areas of interest: (1) family planning, (2) pregnancy, and (3) fertility. We familiarized ourselves with the data by a thorough review of articles that met inclusion criteria and then, based on key conclusions from each article, identified major themes that unified the body of work. The three themes that we have focused on for this review were identified and conceptualized through the principles of Grounded Theory. Important findings from the literature were identified and grouped into themes.

We identified articles that met inclusion criteria from the past twenty years: 1997 to 2016. A majority of the studies were conducted outside of the United States (US), including Argentina, Brazil, India, Iran, Ireland, Kenya, Malawi, Mexico, South Africa, Uganda, and Zimbabwe. Sample sizes ranged from 10 to over 100 participants. In-depth, semistructured interview was the primary method of data collection. Most studies used Grounded Theory as a form of data collection and analysis [11, 12]. Results of these studies were analyzed using descriptive qualitative measures, specifically data coding to generate theory on the lived experiences of the participants. Age ranges of participants varied, with all studies including only men and women of reproductive ages. All studies were primarily focused on heterosexual couples and individuals, with no specific investigations of men who have sex with men (MSM), women who have sex with women (WSW), or any other gender identities or sexual orientations. While women were the majority of participants, men as individuals with HIV or as part of a serodiscordant couple were also included in some of the interviews.

## 3. Results and Discussion

### 3.1. Family Planning

Results from studies discussing family planning (FP) among individuals, couples, and communities noted the importance of childbearing and the right to have children, regardless of the HIV status of the parents in their cultural context. However, HIV stigma, fear of HIV transmission to partner or child, lack of healthcare provider's support, lack of negotiating power for safer sex, and a lack of knowledge or education about safer conception and pregnancy interventions were barriers to having children [9, 13–20].

Among adolescents living with HIV, the desire to possibly have children is high [21]. In one US-based study, 11 youths discussed pregnancy (10 females and one male) and all expressed interest in having a child in the future. Out of the 11, none reported concern about HIV transmission to a partner; they were more concerned about possible vertical transmission [22]. A mixed-methods study on HIV-positive women in Spain interviewed 134 women and found that 49% of the participants expressed a desire to reproduce. This finding was positively associated with (1) lower age (women under 30 had higher reproductive desire than those aged 30–39), (2) having no children, (3) being an immigrant, and (4) not receiving antiretroviral treatment (ART) [23].

Integrating FP services into HIV care can improve contraceptive uptake among women living with HIV in resource-limited settings [24, 25]. However, there is a lack of research examining preconception experiences of HIV-positive men. Findings from in-depth interviews with 21 men living with HIV seeking care at HIV clinics in Kenya suggest that MLWH appear invested in FP. Inclusion of these men in FP decision-making could strengthen both female and male agency in making such decisions [26–28].

When Highly Active Antiretroviral Therapy (HAART) first became available, it was found that the availability of this treatment encouraged patients to have children as their risk of transmitting the virus was substantially decreased, as evidenced by studies in Zimbabwe and Tanzania [29, 30]. Motivation for mothers to follow more intensive and long-term drug regimens often arose from concern for their children's welfare [31]. Similarly, although the idea of assisted reproduction as a way of minimizing the risk of HIV transmission was often initially met with negative reactions, one study, conducted in Kenya, found that once patients learned more about this option, they were interested in it being available in their community [32].

Another South African study explored the attitudes and behaviors of pregnant women and their partners who participated in a behavioral risk reduction intervention. HIV prevalence in the clinics studied ranged from 15 to 40%, and 44 of the 472 study participants were HIV-positive, although less than half had shared that result with their partners. The results suggested that men value and understand the importance of being involved in women's reproductive health. The men interviewed were interested in acquiring information about safe FP with HIV to support their partners [26, 33].

Perceived gender roles around antenatal and delivery care also vary across communities and may play a significant part in the FP process. A study on antenatal and delivery care in Kenya found that male partners of HIV-positive women, while encouraging of their partners to attend antenatal or delivery care, did not generally accompany their wives. Three main barriers were identified, including (1) viewing pregnancy support as a female role, (2) negative healthcare worker attitudes towards male involvement, and (3) antenatal and delivery unit infrastructure that was not friendly for couples [18]. The first barrier was also a common theme in a multisite study in Cameroon, the Dominican Republic, Georgia, and India [34].

Family planning decisions may depend heavily on gendered power dynamics framed by the local sociocultural context. A qualitative study in western Kenya showed a different picture of men's involvement than studies in other communities. Seventy-six in-depth interviews were conducted with 38 couples, of which 22 couples were concordant HIV-positive. Results suggested that FP use signified female promiscuity and infidelity, indicating a need for gender transformative approaches to work within a male-dominated reproductive decision cultural context [35, 36]. A study that compared couples' experiences in a matrilineal and patrilineal community in rural Malawi found that in both communities the husbands' desires were the driving factor for further pregnancies, even when the women were worried about the risks of pregnancy [37].

Timing of childbirth was another significant factor in pregnancy desires and intentions. In-depth interviews with 36 Kenyan heterosexual HIV-serodiscordant couples who had recently conceived found that conception was an urgent matter, largely stemming from a desire for both partners to raise children together while the HIV-infected partner was healthy [38]. Similar findings were seen in a study of HIV-positive women in Tamil Nadu, India. The main factors distinguishing women who wanted to have a child and those who did not were their levels of anxiety about their future health and available family support. Women who decided to have a child did so based on family support, especially when family members offered to take care of the child if one or both of the parents died [39]. A Brazilian study indicated that many women made provisions with their family for the care of their children. Thinking about the possibility of their children becoming orphans made women feel impotent and guilty. Such painful feelings were minimized through defense mechanisms such as compensation, denial, rationalization, and projection [40].

The nature of providers' attitudes towards pregnancy and FP in HIV care was beyond the scope of this paper. However, available studies revealed provider ambivalence regarding childbearing among PLWH and some placed judgment on this population's reproduction in general. Providers recognized the sexual and reproductive rights of PLWH but struggled with concerns about spreading infection [41, 42]. Many providers also believed that reproductive technologies that do not maintain biological and cultural linkage are not acceptable options to their patients [43, 44]. In an Argentinian study, participants reported that healthcare professionals did not acknowledge their reproductive health wishes or provide useful information or referral [17]. Appropriate training is needed to create a more patient-centered approach and to enable providers to better understand the relationship and social realities surrounding their patients' childbearing intentions. As discussed in the pregnancy and fertility sections, the common perception of a provider as inherently powerful shows the dangers of unequal power dynamics between providers and patients. Although a significant number of HIV-positive women intend to have children in the future, few work with providers to safely plan pregnancy [45, 46].

Studies on HIV-related pregnancy counseling suggested that PLWH preferred having HIV-specific services because of better confidentiality and reduced opportunities for unwanted disclosure that could lead to stigma [47]. Interestingly, in one study in Ireland, women diagnosed with HIV during the course of their pregnancy were encouraged by their pregnancy to achieve stability. The diagnosis of HIV, while traumatic, was also a motivating factor [48]. HIV diagnosis during pregnancy and subsequent disclosure to a partner were noted as a common trigger of intimate partner violence among HIV-positive women [43, 49].

### 3.2. Fertility

Understanding how PLWH perceive the significance of maintaining their fertility or having that fertility forcibly taken away is crucial in providing supportive care for these individuals and their partners. To improve the reproductive care for women and men with HIV, it is necessary to explore and understand their fertility constructs and desires within their local context.

We found 12 studies exploring this issue, all of which used qualitative methods. A study in Botswana suggested that cultural norms, specifically the belief that reproduction signifies a full and productive adult, increase risky reproductive and sexual choices. For women, negative themes that emerged included uncertainties about their health futures and the perception of unpleasant experiences in pregnancy [50]. Similarly, a study in Ethiopian women with HIV cited factors such as age, marital length, partner's sexual preferences and views on contraception, duration of HIV diagnosis, and community pressure as factors that influenced fertility desire [51]. Misconceptions about HIV and its effects on fertility were pervasive in a sample of HIV-positive women; most couples stated that unintentional pregnancy occurred because they believed the HIV-positive partner(s) to be infertile [52].

Although partners seemed to make fertility decisions more as a unit, female preferences carried more influence when individual fertility and conception desires differed [53]. This was also true in a study in Durban, South Africa, that showed that women were the dominant decision-makers about fertility, whether they involved their partners or not [54].

Forced sterilization, as a counterpoint to fertility, came up as a theme for women who were HIV-positive [55]. The experiences of HIV-positive women in two South African provinces suggested the rampancy of coercion and pressure on women with HIV to undergo sterilization. One study interviewed twenty-two women who reported their sterilization between 1996 and 2010 without their informed consent or without their knowledge. They cited three issues leading to the subjugation of their reproductive rights: (1) lack of respect for their autonomy, (2) lack of information given about what sterilization entailed, and (3) subtle or overt pressure to sign the consent [56].

Furthermore, a mixed-methods study on HIV-positive women in Latin America found that 23% of the 285 women in the study experienced pressure to undergo sterilization after diagnosis [57]. Healthcare providers who knew their pregnant patients' HIV diagnosis were six times more likely to coerce or force sterilization than with pregnant patients without a known HIV diagnosis. The providers' coercive tactics included misinformation about pregnancy risks and denying medical services needed to prevent vertical transmission, mainly during caesarean delivery. In addition, many of the interviewed women reported losing the rights to choose number of pregnancies or type of contraception after they were diagnosed with HIV. Studies in Mexico and Tanzania cited similar experiences for the women they interviewed. Many of these women were also advised to undergo tubal ligation or abortions [57–61].

### 3.3. Conception and Pregnancy

Wanting and having a child at some point in their lives was a common theme among PLWH in studies worldwide [9, 62]. The desire to have a child versus intentional pregnancy was mediated by sociocultural and health perceptions and beliefs about conception, pregnancy, and postnatal life with children. Having children was seen as a reason to have hope in the future [63].

A study on pregnancy intentions in South Africa and Malawi among HIV-positive women and their partners, seroconcordant and serodiscordant, found that a partner's expectations for pregnancy, finances, and family composition were key factors in negotiating pregnancy desires. The majority of participants did not actively plan for a pregnancy and challenged the notion that reproductive decisions are always conscious actions [64]. Women's conscious decisions, cited in another study, were based on their judgment of the positive aspects of motherhood versus the negative pressures of social stigma [65].

A sample of ten individuals in serodiscordant relationships interviewed in Northern Ireland reported a willingness to accept the biological risk of infection through unprotected sex in order to meet their desires to conceive without medical interventions, which included but were not limited to ART. This decision-making was also influenced by negative experiences with healthcare providers who were unwilling to support their HIV-positive patients' attempts to conceive [53].

The experiences of a repeat pregnancy are also important in how PLWH view having additional children. Akelo et al. interviewed 40 HIV-positive Kenyan women about their experiences of repeat pregnancy [66]. Qualitative analysis revealed concerns about modern contraception methods as having negative side effects that “ruined the womb.” Having the “right” number of children was another important theme for these women and was reinforced by religious leaders, family, and the broader community. A multisite study conducted in South Africa found that pregnancy desires are motivated by whether or not the participant has had any children with his/her current partner rather than by the number of living children. Interviews conducted were with both men (37%) and women (63%) [67].

The presence of HIV-related stigma inevitably affects the women's experience of pregnancy and care. Positive experiences of pregnancy care in Ireland were associated with empathy and understanding of unique needs, continuity of care, and normalizing pregnancy with HIV as much as possible [4, 68]. A literature review of studies focused on the pregnancy decisions of HIV-positive women showed that neonatal outcomes often received more attention than the health of the HIV-positive mother pre-, peri-, and postnatally. It also highlighted the impact of social stigma, discrimination, and, in many countries, the criminalization of HIV transmission that pregnant HIV-positive women must navigate throughout their pregnancies [69].

Having an HIV diagnosis also complicates abortion intentions and decisions. Women living with HIV in Cape Town, South Africa, reported stigma for both becoming pregnant and terminating a pregnancy. This stigma was both self-imposed and from external sources, including family members, partners, community members, and healthcare providers. Despite South Africa's liberal abortion law, abortion is potentially more stigmatizing than pregnancy for women living with HIV [70, 71]. In countries where abortion laws are more restrictive, like Nigeria and Zambia, community attitudes heavily favor continuing childbearing over abortion for women living with HIV [72].

For women living with HIV, their HIV status is an important part of the decision-making around pursuing an abortion. Other factors considered in terminating the pregnancy included stage of life when pregnancy occurred and the level of support from partners and/or families [73, 74].

## 4. Discussion

There is a small but rich pool of qualitative studies that provide some insight into how an HIV diagnosis may complicate beliefs, perceptions, and behaviors related to reproductive health. The findings described suggest a need for better understanding of the complex psychosocial milieu surrounding fertility, family planning, and reproduction for PLWH.

Because there were no qualitative studies of reproductive beliefs of PLWH who identify as LGBTQ^*∗*^, the findings in this paper reflect only heterosexual PLWH. Research is needed to build an understanding of reproductive health perceptions for PLWH who identify as LGBTQ^*∗*^ in order to best meet these patients' needs.

This review highlights the importance of developing and leveraging trusted provider and community-based relationships and assessing patients' beliefs and values in a sociocultural context, to ensure clear, consistent, and relevant communications. Based on the available literature, PLWH may benefit from more intentional integration of reproductive health in existing HIV care provision programs. Additionally, perceptions, beliefs, and preferences around fertility, family planning, conception, pregnancy, and termination rely heavily on gender roles and the sociocultural context in which the patient lives. The necessary next step will be to identify, implement, and evaluate policies and practices addressing reproductive health in HIV populations that are culturally congruent and accessible.

Qualitative research is a valuable tool to begin understanding patients' perspectives on reproductive health and to generate hypothesis-driven quantitative research to support the reproductive health for PLWH. This review illustrates the value of qualitative narratives in building a more comprehensive understanding of reproductive health perceptions for PLWH. Integrating qualitative interviews into components of HIV research on reproductive health will move clinical care, policy, and interventions forward, as well as providing an opportunity to empower patients to talk about their experiences. It is crucial to produce further published research demonstrating that the patient is the expert in this area of healthcare.
